# Systematic analysis of nutrient-microbiome interactions and their effects on host phenotypes in *Drosophila*

**DOI:** 10.1128/mbio.02480-25

**Published:** 2025-11-05

**Authors:** Yi-Ting Hung, Shu-Jen Tuan, Adam Chun-Nin Wong

**Affiliations:** 1 Department of Entomology, National Chung Hsing University, Taichung, Taiwan, Republic of China; 2 Department of Entomology and Nematology, University of Florida, Gainesville, Florida, USA; 3 Genetics Institute, University of Florida, Gainesville, Florida, USA; University of California Irvine, Irvine, California, USA

**Keywords:** *Drosophila melanogaster*, microbiome, metabolism, nutrition, geometric framework, *Acetobacter*, *Levilactobacillus*, locomotion, sleep

## Abstract

Diet and the gut microbiome are interconnected forces shaping host physiology and behavior. However, disentangling their independent and combined effects on phenotypic variation remains a major challenge. Here, we systematically assessed 120 combinations of nutrient and microbiome conditions to evaluate their effects on *Drosophila melanogaster* phenotypes. By varying dietary yeast-to-sucrose ratios (Y:S) and concentrations, coupled with five defined microbiome configurations, we disentangled the complex diet-microbiome interplays on metabolic, reproductive, and behavioral traits. Our analyses show that abundances of the major gut bacteria *Acetobacter pasteurianus* and *Levilactobacillus brevis* are differentially shaped by macronutrient components. *A. pasteurianus* exhibits a linear positive association with dietary yeast, whereas *L. brevis* displays a non-linear response driven more strongly by the yeast-sucrose ratio. In the host phenotypes, body protein and triglyceride levels are primarily determined by the nutrient environment, while the microbiome exerts a more notable influence on glucose metabolism. Microbial species exhibited context-dependent effects: *A. pasteurianus* facilitated protein assimilation and weight gain under high-yeast conditions, whereas co-colonization with *L. brevis* synergistically amplified fecundity. Critically, microbiome influences on locomotion and sleep varied by dietary Y:S, which helps explain discrepancies in prior studies that did not account for dietary variability when assessing microbiome-behavior relationships. By conceptualizing diet as a multidimensional nutrient environment, our study introduces a framework to better predict how dietary and microbial variation shape host phenotypes. These findings highlight that microbial contributions to host phenotypes are not fixed but emerge from interactions with the nutritional environment, thus advancing a more ecologically realistic framework for studying diet–microbiome relationships.

**IMPORTANCE:**

The interplay between diet and the gut microbiome is fundamental to shaping host physiology and behavior; however, their interactions remain poorly understood. Most studies treat diet as a single-dimensional variable (e.g., high-fat or high-sugar), overlooking the complexity of nutrient balance and density. This oversimplification neglects how diet and microbes function as an integrated system. This study addresses this gap by testing 120 different nutrient-microbiome combinations in *Drosophila melanogaster*, systematically varying yeast and carbohydrate levels and microbiome configurations. Our results show that dietary nutrient composition drives body protein and fat storage, whereas the microbiome plays a notable role in glucose metabolism and buffers against excess fat accumulation. Microbial effects on reproduction, locomotion, and sleep depend on nutrient composition, and our model reveals specific diet-microbiome patterns driving these outcomes. By treating diet as a dynamic, multidimensional factor, we provide a novel, ecologically relevant framework for understanding how diet and microbiome shape host.

## INTRODUCTION

The gut microbiome plays a multifaceted role in shaping host physiology and behavior by modulating nutrient allocation, energy balance, and metabolic homeostasis (1–3). Reciprocally, diet composition can alter the structure and function of the gut microbiome (4–6), with microbial effects on host phenotypes varying depending on the nutrient conditions (7, 8). These dynamic, bidirectional interactions not only impact metabolic health but also broader evolutionary and ecological traits, such as tolerance to abiotic stressors (9–11), pheromone synthesis (9, 12), and reproductive success (13, 14). However, despite the recognized importance of diet–microbiome crosstalk, our understanding of how specific microbial taxa interact with nutrient environments to influence host phenotypes remains limited.

The geometric framework for nutrition has been instrumental in elucidating how variation in dietary macronutrients, particularly protein and carbohydrate, drives life-history trade-offs across animal species (15–17). However, the potential role of the microbiome as a nutrient-responsive modulator of these outcomes has been largely overlooked in nutritional geometry studies. Conversely, studies of the microbiome’s role in host physiology often treat diet as a one-dimensional variable (e.g., “high-fat” or “high-sugar”), failing to capture the complexity of nutrient combinations. These parallel gaps have limited our ability to predict or explain how microbial influences manifest across nutrient environments. Key questions remain unresolved: under which dietary conditions do microbes exert the most significant effects? Which host traits are shaped by microbes consistently across diets, and which depend on specific nutrient contexts? And do microbial contributions operate additively, synergistically, or antagonistically with diet in determining specific host outcomes?

To address these gaps, we integrated microbiome variation into a nutritional geometry framework. Building on foundational work in *Drosophila melanogaster* demonstrating that gut microbes modulate host developmental and metabolic responses to dietary yeast and carbohydrate through mechanisms including B-vitamin provisioning, enhanced protein nutrition, and modulation of lipid storage (8, 18), we comprehensively dissect diet–microbiome interactions across a multidimensional nutrient space. Specifically, we generated 120 unique diet-microbiome combinations by pairing five distinct microbiome treatments of mono- and dual-associations with two dominant *Drosophila* gut bacteria, with 24 diets that systematically varied in yeast-to-sucrose ratio (Y:S) and concentration. Yeast and sucrose served as proxies for protein and carbohydrate, respectively, in line with previous applications of the geometric framework to *Drosophila* nutrition studies (16, 19). This experimental design enables us to quantify the relative and interactive effects of microbes and nutrients on host traits, identify nutrient conditions in which microbial effects are amplified or diminished, and map how individual microbial taxa respond to nutrient variation. In addition to metabolic traits, we examined locomotion and sleep, behavioral traits that are increasingly recognized as being modulated by both dietary and microbial factors (20–22).

Our results reveal that macronutrient composition is the dominant driver of host nutritional and reproductive traits. Specific macronutrient components can differentially influence the abundance of gut microbes, as evidenced by a strong positive linear association between dietary yeast and *A. pasteurianus*, whereas no such relationship was observed for *L. brevis*. We also identified distinct microbial roles dependent on the nutrient environment. For example, *Acetobacter pasteurianus* specifically enhanced protein assimilation under high-yeast conditions, whereas co-colonization with *Levilactobacillus brevis* synergistically amplified fecundity. For behavior, diet–microbiome interactions emerged as the strongest explanatory factor. The influence of microbiome on these behaviors varied across Y:S conditions, providing a plausible explanation for the discrepancies seen in previous studies that did not account for dietary context.

Taken together, our study demonstrates that microbial effects on host phenotypes are not fixed properties of specific taxa but emerge from interactions with the host’s nutrient environment. Macronutrient variability not only shapes microbial abundance but also modulates the functional consequences of specific taxa on host physiology and behavior. This framework provides a more ecologically realistic approach for predicting context-dependent microbial contributions to host phenotypes.

## MATERIALS AND METHODS

### Fly culture and microbiome manipulations

*Drosophila melanogaster* (Canton-S, *Wolbachia*-free) used in our experiments were laboratory wild-type stocks obtained from the Handler lab (USDA-ARS). The flies were reared at 25°C on an autoclaved yeast-sugar diet (comprising 50 g L^−1^ sugar [Walmart, Bentonville, AR, USA], 100 g L^−1^ Brewer’s yeast [MP Biomedicals, Solon, OH, USA], 12 g L^−1^ agar [Genesee Scientific, San Diego, CA, USA], and preservatives comprising 0.04% phosphoric acid [Thermo Fisher Scientific, Waltham, MA, USA], and 0.42% propionic acid [Sigma-Aldrich, St. Louis, MO, USA]), under a 12 h: 12 h light:dark photoperiod regime. *Acetobacter pasteurianus* and *Levilactobacillus brevis* were isolated from dissected *Drosophila* guts by plating gut homogenates on the MRS agar (Research Products International Corp, Mt Prospect, IL, USA) and incubating at 30°C for 48 h. Bacterial identification was performed by PCR followed by Sanger sequencing of the 16S rRNA gene, using primers 27F (AGAGTTTGATCMTGGCTCAG) and 1522R (AAGGAGGTGATCCAGCCGCA). The resulting 16S rRNA gene sequences have been deposited in NCBI GenBank under accession numbers PQ451486 and PQ451487.

Axenic flies were derived from dechorionated eggs as described in Ridley et al. (23). For egg dechorionation, eggs deposited overnight by mated females were washed in sterile water, then immersed in 0.75% sodium hypochlorite solution for 2 min, repeated three times. Afterward, the eggs underwent two consecutive rinses in sterile water before being transferred to the sterile diet within a biosafety cabinet. To produce gnotobiotic flies, the *Drosophila*-associated bacteria were cultured in the MRS broth (Research Products International Corp) at 30°C using two-position snap-cap tubes. We followed the method outlined by Koyle et al., gnotobiotic flies associated with a single species, either *A. pasteurianus* or *L. brevis*, by adding 50 µL bacterial suspension at 10^7^ colony-forming unit (CFU) mL^−1^ density onto each food vial (30 mm diameter) containing the dechorionated eggs (24) . For the two-species association flies, 25 µL of bacterial suspension was added for each bacterium. To confirm the microbiome status of the flies, the diet rearing the axenic and gnotobiotic flies was plated onto MRS and LB agar plates. The plates were incubated at 32°C for three days, after which they were examined for any contamination and colony morphology corresponding to *Acetobacter* and *Levilactobacillus*. Conventional flies were raised from the same batches of eggs used for producing axenic and gnotobiotic flies by directly transferring them to the sterile diet.

### Diet preparation

**Fig 1:**
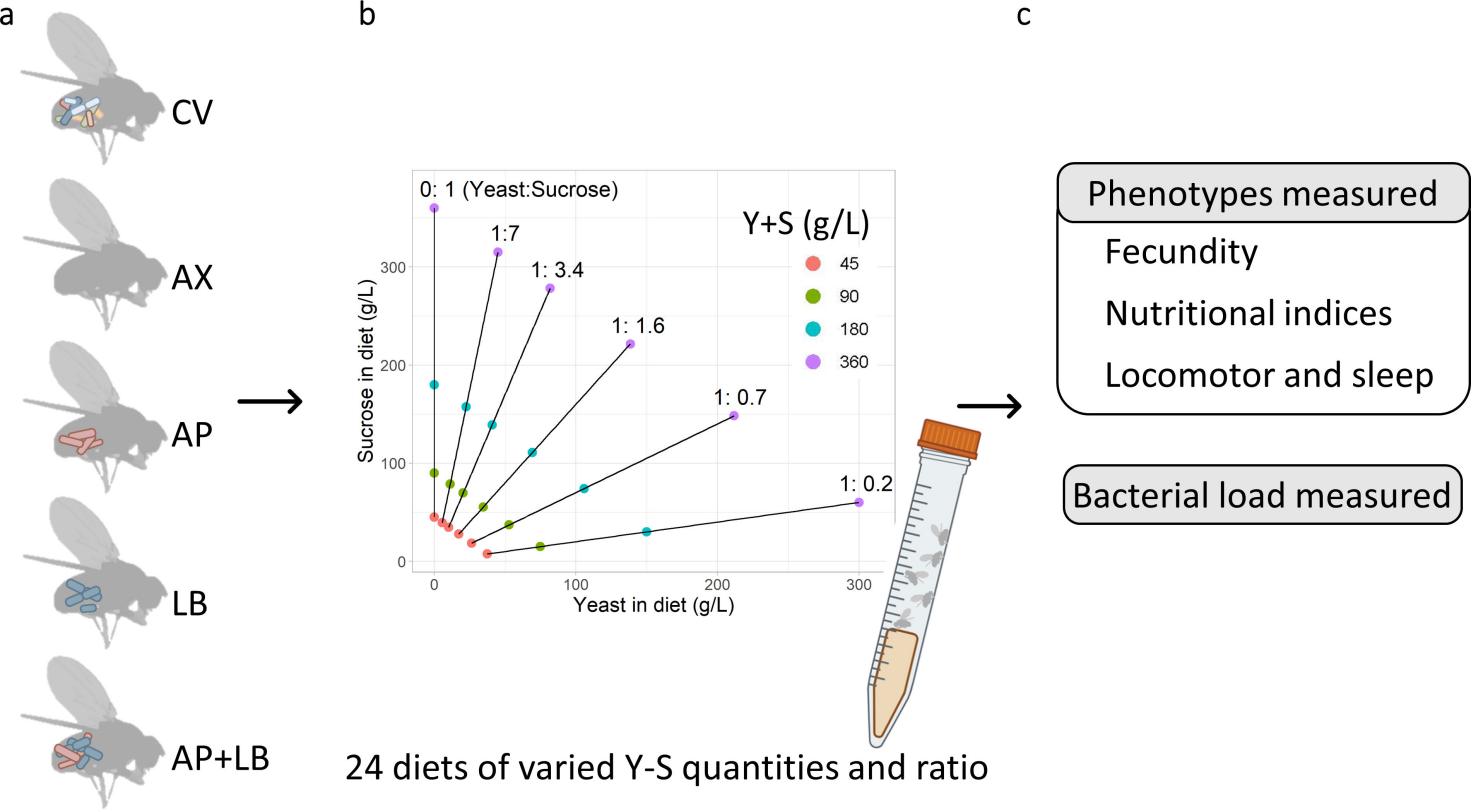
Experimental scheme. (**a**) Five microbiome treatment groups were raised on a regular yeast-sugar diet. (**b**) Newly emerged female flies were conditioned on one of the 24 diets of varying yeast (Y) to sucrose (S) ratios and a total Y + S level. The nutrient radials (solid lines) originating from the origin indicate six Y:S ratios (from left to right: 0:1, 1:7, 1:3.4, 1:1.6, 1:0.7, 1:0.2). Each radial includes four Y + S levels (45 g/L, 90 g/L, 180 g/L, 360 g/L). (**c**) Bacterial CFUs, nutritional indices, fecundity, locomotion, and sleep were measured after the diet conditioning. CV: conventional flies; AX: axenic flies; AP: gnotobiotic flies with *A. pasteurianus*; LB: gnotobiotic flies with *L. brevis*; AP + LB: gnotobiotic flies with both *A. pasteurianus* and *L. brevis*. The figure was partially created with BioRender.com.

Following the protocol outlined in Lee et al. (16), a total of 24 diets differing in their yeast (Y) to sucrose (S) ratios and combined Y + S concentrations were prepared by autoclaving (Fig. 1a). The composition of the diets varied in yeast powder (Y) and sucrose (S) content. Specifically, six yeast-to-sucrose (Y:S) ratios were utilized, including 0:1, 1:7, 1:3.4, 1:1.6, 1:0.7, or 1:0.2, resulting in corresponding Y:S ratios of 0:1, 1:16, 1:8, 1:4, 1:2, and 2:1, respectively. Four final concentrations of Y + S were prepared, specifically 45, 90, 180, or 360 g L^−1^.

### Experimental protocol

Five groups of flies with different microbiome compositions were prepared for fecundity, nutritional, behavioral, and colony-forming units (CFU) assays: CV (conventional), AX (axenic), AP (gnotobiotic flies with *A. pasteurianus*), LB (gnotobiotic flies with *L. brevis*), and AP + LB (gnotobiotic flies with both *A. pasteurianus* and *L. brevis*) (Fig. 1a). Flies in the preadult stage were raised on the regular yeast-sugar diet. Newly emerged flies were first transferred to a new yeast-sugar diet for 24 h for mating. Then, five pairs of flies were transferred to each of the 24 experimental diets for a 5-day dietary pretreatment (Fig. 1b). For both bacterial load and nutritional index measurements, five female flies were then collected per diet-microbiome treatment, with five replicates for each treatment (Fig. 1c). For the fecundity and behavioral assays, a single female fly was used per diet-microbiome treatment, also with five replicates (Fig. 1c).

### Colony-forming units (CFU) assay

The microbiome status in each diet-microbiome treatment was confirmed using a CFU assay according to Dodge and Ludington (25). Following diet pretreatment, samples of five female flies were collected and surface sterilized with 70% ethanol and sterile water. The surface-sterilized flies were then homogenized by bead beating in 125 µL of MRS medium in Lysing Matrix D tubes containing 1.4 mm Zirconium-Silicate spheres (MP Biomedicals). The resulting mixture was diluted to 1,000 µL with MRS and subsequently serially diluted to a ratio of 1, 8, 64, and 512. The diluted mixture was then plated onto MRS agar in single-well plates. The plates were incubated at 32°C for 48 h before counting the colonies. The equation used to calculate the number of CFUs per fly is as follows: E = C × D/P × V/F, where E = CFU per fly, C = number of colonies counted, D = dilution, P = µL plated, V = volume of fly homogenate, and F = number of flies homogenized.

### Nutritional indices

Pools of five female flies were frozen and weighed on a microbalance (Cahn C-35 microbalance [±1 µg], Orion Research, Boston, MA, USA). The samples were then homogenized with 100 µL of matrix D beads (MP Biomedicals) using a bead beater (Precellys Evolution, Bertin Technologies, Montigny-le-Bretonneux, France) at 8,000 rpm for 2 cycles of 15 s each in 125 µL ice-cold TET buffer (autoclaved 35 mM Tris, 25 mM KCl, 10 mM MgCl_2_, 1 mM EDTA, 0.1% Triton X-100, pH = 8), followed by centrifugation at 15,000 × *g* at 4°C for 3 min. The protein level of the flies was determined using 50 µL of the supernatants diluted 10-fold. Total protein was measured using the DC Protein Assay kit (Bio-Rad, Hercules, CA, USA) with a microplate spectrophotometer (800 TS plate reader, Agilent BioTek Inc., Santa Clara, CA, USA), following the manufacturer’s instructions. The remaining supernatants were heated to 72°C for 30 min for glucose and triglyceride measurements. Glucose and triglyceride levels were assessed using the Glucose Assay Kit (Sigma-Aldrich) and the InfinityTM Triglycerides liquid stable reagent (Thermo Fisher Scientific), respectively. The nutritional indices were normalized to weight and expressed as μg mg^−1^ body mass.

### Fecundity performance

Following dietary pretreatment (as described in Section 2.3), one female and two males were transferred to vials containing 5 mL colored diets (Green food color, Gel spice co., Bayonne, NJ) with the same Y:S ratios as the pretreatment diets. The added coloring was used to visualize the eggs laid on the diet surface. The flies were transferred to fresh vials daily, and the number of eggs laid was recorded for 2 days. Fecundity was assessed by combining the data collected on days 1 and 2 of the assay period.

### Behavioral indices

Sleep behavior was measured using the Drosophila Activity Monitor System (DAM5H, Trikinetics, Waltham, MA, USA). Female flies were individually loaded into Trikinetics single-fly tubes (65 × 5 mm glass tube) containing a small amount of the 24 diets at one end and a small cotton plug at the other end. The tubes were then inserted into the activity monitors with the food positioned adjacent to beam number “1.” The monitors were placed at room temperature on a 12 h light-dark cycle. Data were collected by the DAMSystem3 software (Trikinetics), wherein move counts, activity counts, and positions were recorded once per minute throughout the monitoring period. All flies were provided with a minimum of 24 h to acclimatize to the DAM5H. Subsequently, data extraction started for a period of 72 h, starting from the following lights-on event.

Analysis of the data obtained from DAM5H on move counts, activity counts, and positions was conducted using R (version 4.3.2). The corresponding analysis scripts can be accessed via the following link: https://github.com/Tiffany9583/Trikinetic_DAM5H.git. The following behavioral parameters were obtained from the analysis: average counts, the frequency of a fly passing through the red beams per day; moving distance, the total distance traversed by a fly, determined by calculating the distance between positions recorded by the monitor; and average sleep duration, the minutes a fly sleeps per day. Sleep was defined as a period of 30 min or more with consecutive minutes of inactivity, as outlined in Chowdhury et al. (26). The light and dark phases during the recording were determined by the light sensors on the monitor.

### Modeling and data analysis

#### A Bayesian approach for generalized linear mixed models

To determine whether the microbiome or diet is the dominant factor influencing nutritional indices and behavioral parameters, we used a Bayesian generalized linear mixed model (GLMM). In this model, microbiome, diet, and microbiome × diet were treated as random effects, allowing us to assess their individual contributions to phenotypic variation (Fig. S1a). By partitioning the variance explained by each random effect, we can quantify and compare the relative influence of microbiome and diet on the observed *Drosophila* phenotypic traits.

The response variable *y*_trait_ represents the observed phenotypic traits assessed in this study, including body weight, fecundity, nutritional indices, activity, and sleep duration in *Drosophila*. The model was specified as:


$$y_{trait}=\beta_{0}+\mu_{rep:micro}+\mu_{rep:diet}+\mu_{micro\times diet}$$

where *β*_0_ is a global intercept representing the overall mean response across all conditions, and *u*_rep:micro_ is a random effect accounting for variation among replicates within each microbiome treatment group. Microbiome groups include flies treated with *A. pasteurianus*, *L. brevis*, a combination of *A. pasteurianus* and *L. brevis*, as well as conventional and axenic groups. The term *u*_rep:diet_ is a random effect capturing variability among replicates nested within 24 diet treatments, whereas *u*_micro× diet_ captures the interaction effect between microbiome treatment and diet. The model involved 4,000 posterior samples processed via “Stan” function from the “rstanarm” package version 2.32.1 (27).

#### Estimating the contribution by a linear model

To identify which specific sub-level factors (e.g., dietary yeast, sucrose, Y:S ratio, dietary concentration levels, CFUs of *A. pasteurianus* and *L. brevis*, and their interaction) most strongly influence the traits, we employed multiple linear regression (MLR) analysis to evaluate the relationship between the trait variable and these factors (Fig. S1b). To assess the relative importance of each factor, defined as its unique contribution compared to other variables in the model, we applied methods that go beyond traditional statistical metrics. Conventional measures such as correlation coefficients, standardized regression coefficients, and *P*-values are often inadequate when predictors are correlated, as they do not account for shared variance. To address this limitation, we used the Lindeman, Merenda, and Gold (LMG) method (28), which estimates the average contribution of each predictor across all possible orderings of regressors by decomposing the model’s total R². In addition, we applied exploratory bootstrap confidence intervals to the importance measures to improve the robustness and interpretability of the results (29).

The response variable *y*_trait_ represents the observed phenotypic traits assessed in this study, including body weight, fecundity, nutritional indices, activity, and sleep duration in *Drosophila*. The linear regression model was specified as:


$$y_{trait}=\beta_{0}+\beta_{yeast}+\beta_{sucrose}+\beta_{ratio}+\beta_{conc\times ratio}+\beta_{AP}+\beta_{LB}+\beta_{AP\times LB}$$

where *β*_0_ is a global intercept. The terms *β*_yeast_, *β*_sucrose_, and *β*_ratio_ represent the fixed effects of dietary yeast content, sucrose content, and Y:S ratio, respectively. The interaction term *β*_conc×ratio_ accounts for the effect of dietary concentration levels interacting with the Y:S ratio. The coefficients *β*_AP_, *β*_LB_ capture the effects of CFUs with *A. pasteurianus* and *L. brevis*, respectively, whereas *β*_AP×LB_ models the interaction effect of co-colonization with both bacterial species. The relative importance of each factor, along with bootstrap confidence intervals, was estimated using the “relaimpo” package version 2.2.7 (29).

#### Geometric framework for nutrition

The geometric framework for nutrition (GFN), a state-space model representing nutrition in a multidimensional nutrient space (15), was used to interpret systematically varied dietary yeast and sucrose levels. Coordinates within this space captured the effects of specific nutrient combinations on tested traits. Building on this framework, generalized additive models (GAMs) with thin-plate splines were applied to analyze nonlinear relationships between dietary composition and observed traits. The yeast and sucrose contents of the diets (g L^−1^) were fitted as a two-dimensional smoothed predictor. The smoothing parameter is chosen by generalized cross-validation. Predictions from GAMs were generated for a representative coordinate matrix from the experimentally captured component of the nutrient space. These predictions were then visualized as nutrient surfaces to fully show the effects of dietary macronutrients on fecundity, nutritional, and behavioral indices. The response surface was generated with the “fields” package version 15.1 (30).

Residual distributions for the models were validated using the “simulateResiduals” function from the “DHARMa” package version 0.4.6 (31). Pearson’s correlation coefficients between the indices were calculated using the “corrplot” package version 0.92 (32). Scatterplots of bacterial CFU trends were fitted with the loess function and visualized using the “ggplot2” package version 3.5.1 (33). The above analyses and visualizations were performed using R version 4.3.2. The files containing phenotypic observations and bacterial load data are available at https://github.com/Tiffany9583/Nutrient-microbiome-interactions-on-Drosophila.

## RESULTS

To investigate how diet, microbiome, and their interactions shape key nutritional, fecundity, and behavioral traits, we conditioned flies from five distinct microbiome composition groups on 24 diets varying in yeast and sucrose content. Four of the groups were associated with bacteria, whereas one group was microbe-free (AX group). The bacteria-associated groups included flies mono-associated with *A. pasteurianus* (AP group), *L. brevis* (LB group), both species (AP + LB group), and conventionally reared flies (CV group).

To investigate the general effects of diet and microbiome on *Drosophila* phenotypes, we first employed Bayesian hierarchical models to estimate the proportion of variance attributable to broad microbial and dietary categories. This analysis allowed us to identify whether the microbiome, diet, or their interaction (microbiome × diet) was the dominant factor influencing each trait (Fig. S1a).

Next, we disaggregated the diet and microbiome into specific sub-level factors—including dietary yeast, sucrose, yeast-to-sucrose ratio, total diet concentration, colony-forming units (CFUs) of AP and LB, and their interaction—to evaluate their individual contributions in explaining variation in nutritional and behavioral traits. Using multiple linear regression, we decomposed the total model *R*^2^ to determine which sub-level factors most strongly influenced each phenotypic trait. This approach enabled us to identify the most impactful components of diet and microbiome that drive variation in *Drosophila* nutritional and behavioral traits (Fig. S1b).

To visualize the multidimensional trait variation across the systematically designed diet-microbiome combinations, we generated response surface heatmaps. These heatmaps captured how traits varied across dietary and microbial gradients. Although the microbiome exerted relatively weaker effects on nutritional and behavioral traits, the impact of diet varied distinctly across different microbiome conditions. To further explore these patterns, Pearson’s correlation analysis was performed to assess linear relationships among traits within each microbiome group (Fig. S1c). Additionally, given the potential influence of nutritional profiles on behavioral outcomes, we also examined correlations between nutritional and behavioral traits under each microbiome condition (Fig. S1d). Notably, a significant Pearson’s correlation indicates that a relationship exists, but does not reflect the strength or practical effect size of that relationship. Together, these complementary approaches provided a comprehensive understanding of how diet and microbiome jointly shape phenotypic variation, offering insights into both the direction and magnitude of their effects.

### Bacterial loads of *A. pasteurianus* and *L. brevis*

**Fig 2:**
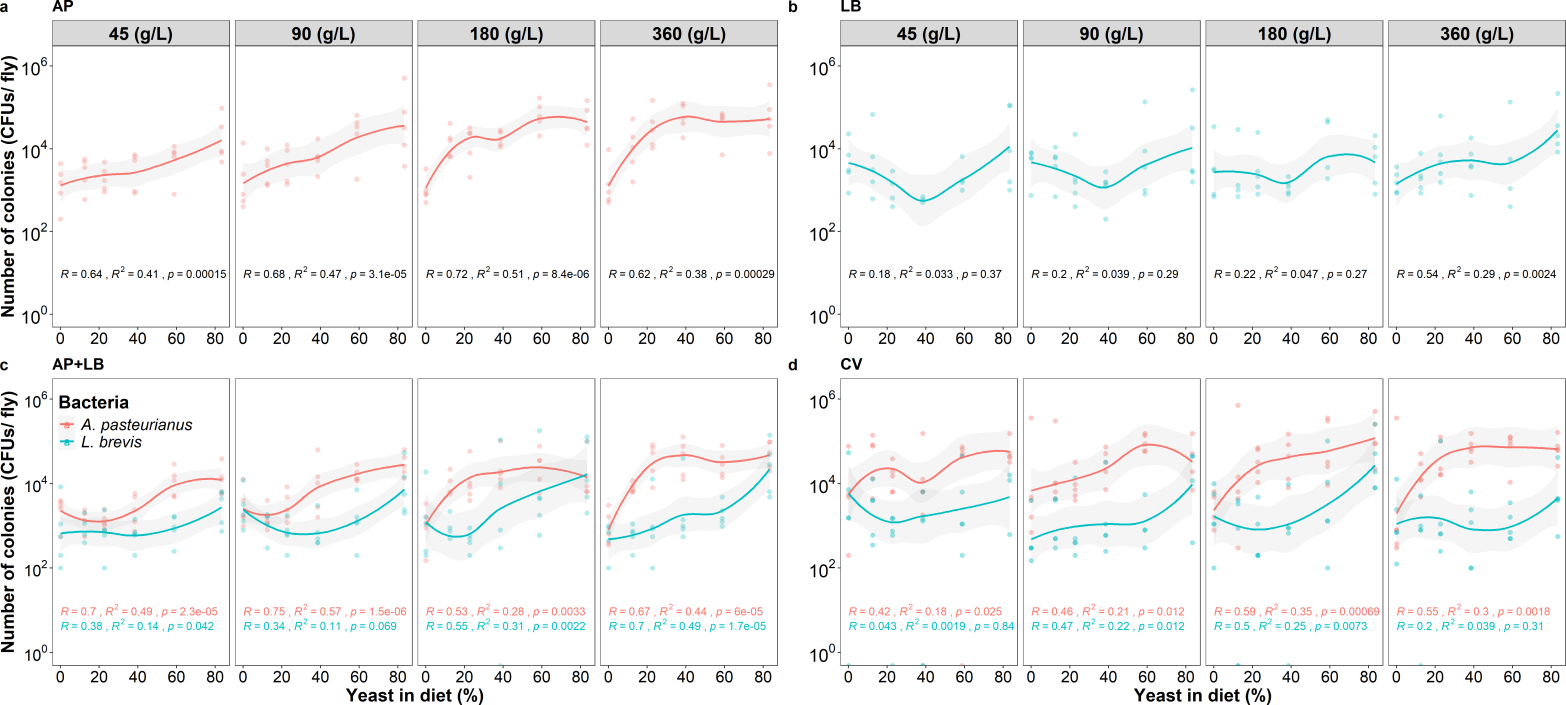
Fit plot for CFUs of *A. pasteurianus* and *L. brevis* in (**a**) the gnotobiotic flies with *A. pasteurianus* (AP)*,* (**b**) gnotobiotic flies with *L. brevis* (LB), (**c**) gnotobiotic flies with two species, *A. pasteurianus* and *L. brevis* (AP + LB), and (**d**) the conventional flies (CV) fed on diets varying in yeast content. The red lines indicate CFUs of *A. pasteurianus*. The blue lines indicate the CFUs of *L. brevis*. Lines are fitted using locally estimated scatterplot smoothing. The gray shaded area represents the 95% CI. *R*, the correlation coefficient; *R*^2^, the coefficient of determination. *P*: *P*-value of the correlation coefficient.

Our findings based on fly colony-forming units (CFUs) across the 24 dietary conditions demonstrate that the abundances of the two dominant gut microbiome members, *A. pasteurianus* and *L. brevis*, are influenced by dietary yeast and sucrose levels in distinct ways. In the AP group, *A. pasteurianus* loads increased with higher dietary yeast, reaching a peak of ~10^5^ CFU per fly (Fig. 2a). Similar trends were observed in the AP + LB (Fig. 2c) and CV (Fig. 2d) groups. This relationship was supported by linear models that showed a significant positive association between dietary yeast and AP CFUs (*P* = 0.0068, Table S1) and that dietary yeast explains the majority of variance in AP CFUs (61.5%, Table S2). These results suggest that the abundance of *A. pasteurianus* is driven by the availability of dietary yeast, which provides protein and other micronutrients, and this effect remains consistent regardless of the presence of *L. brevis*.

In contrast, *L. brevis* loads displayed a more complex, nonlinear pattern. For diets with lower yeast and sucrose concentrations (45, 90, and 180 g L^−1^), *L. brevis* levels initially declined at lower yeast levels, then rose with increasing yeast concentrations, reaching a peak of ~10^4^ CFU per fly, resulting in a V-shaped trend (Fig. 2b). At the highest yeast and sucrose concentration (360 g L^−1^), *L. brevis* loads also peaked at the highest yeast percentage but did not show the same decline at lower yeast levels. Linear models did not detect significant individual linear effects of yeast or sucrose on *L. brevis* abundance (Table S1); instead, the Y:S ratio accounted for the greatest proportion of variation (55.6%, Table S2), consistent with the observed nonlinear response. This suggests that *L. brevis* growth is modulated by the balance of yeast and carbohydrate rather than by yeast alone.

We also plotted bacterial loads with respect to dietary sucrose levels, shown in Fig. S2. The trends observed across varying sucrose levels were largely inverse to those seen with dietary yeast, as expected, given that only these two nutrient components were varied in the diets. For example, *A. pasteurianus* loads decreased with increasing sucrose, consistent with its positive association with yeast. The bacterial load data from the AP + LB and CV groups demonstrate that *A. pasteurianus* maintained a consistently higher abundance compared with *L. brevis* across the diverse dietary conditions tested. Furthermore, there were no statistically significant differences in the bacterial loads between the co-culture groups and their corresponding mono-associated groups. This observation suggests that under the experimental parameters of this study, the two gut symbionts did not exhibit notable competitive or mutualistic interactions.

### Microbiome × diet interactions on nutritional profiles

**Fig 3:**
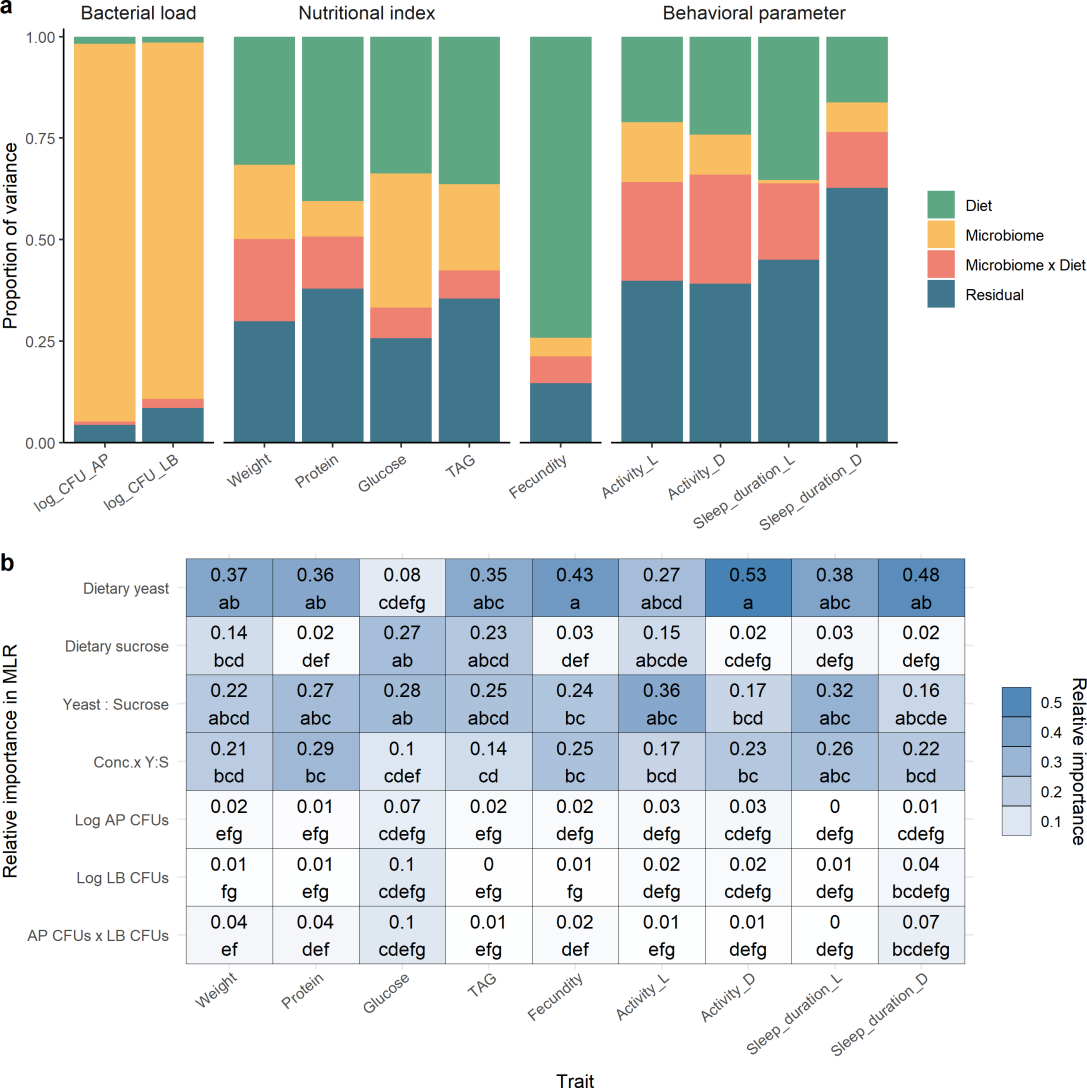
(**a**) The proportion of variance explained by diet, microbiome, and their interaction for the different nutritional indices and behavioral parameters studied. Each bar represents the contribution of diet and microbiome to the dietary yeast and sucrose variance of the different variables from Bayesian hierarchical models. Confidence intervals and Monte Carlo standard error are provided in Table S3. (**b**) Relative importance of predictors in a multiple linear regression (MLR) model explaining the effects of dietary yeast, sucrose, and bacterial loads on nutritional indices and behavioral parameters, based on decomposed *R*² values. Conc. × Y:S: the interaction between yeast-to-sucrose ratio and total nutrient concentration. Confidence intervals and the total proportion of variance explained by each model are provided in Table S4. Different letters indicate statistically significant differences within each column, determined by non-overlapping confidence intervals at *P* < 0.05. Log AP CFUs and Log LB CFUs: log-transformed bacterial loads of *A. pasteurianus* and *L. brevis*, respectively. Activity: counts a fly passing through the red beams; Sleep duration: minutes a fly sleeps per day; L: light phase (daytime); D: dark phase (nighttime).

Nutritional profiles, including body weight, protein, glucose, and triglyceride (TAG) levels, were significantly influenced by both diet and microbial composition. The Bayesian hierarchical model revealed that the diet, microbiome, and their interactions accounted for 32%, 18%, and 20% of the variance in fly body weights, respectively, indicating that all three factors exerted comparable moderate effects on this trait (third bar in Fig. 3a). However, diet exhibited a stronger influence than the microbiome on both protein and TAG levels, with effect sizes 4.6 and 1.7 times greater, respectively. For glucose levels in flies, the effects of diet and microbiome were similar in magnitude, explaining 34% and 33% of the variance, respectively. The effects of microbiome × diet interactions were relatively small on protein, glucose, and TAG levels, accounting for 13%, 7%, and 7% of the variance, respectively (Fig. 3a). Relative importance analysis using multiple linear regression (MLR) models further elucidated the distinct contributions of dietary macronutrient composition and bacterial community structure to host nutritional profiles (the first to fourth columns in Fig. 3b. Darker blue indicates higher relative importance). Dietary yeast content was found to significantly impact body weight, protein, glucose, and TAG levels, whereas dietary sucrose primarily affected glucose and TAG, but not protein levels in the flies. The Y:S ratio consistently exhibited significant effects across all nutritional traits. However, when the interaction between the Y:S ratio and total nutrient concentration was included, its relative importance for explaining glucose levels decreased, suggesting that the nutrient ratio influences glucose metabolism independently of the overall dietary concentration.

In contrast to dietary effects, only glucose levels were notably influenced by the loads of *A. pasteurianus*, *L. brevis*, and their interaction, with relative importance values of 7%, 10%, and 10% of the model’s explained variance, respectively (the third column in Fig. 3b). Although smaller than the effects of dietary sucrose and the Y:S ratio, these findings suggest that microbial load also contributes to glucose metabolism in *Drosophila*. In addition, when comparing the individual impacts of the two bacterial species, *A. pasteurianus* load had a broader effect, more strongly influencing body weight, glucose, and TAG levels, whereas *L. brevis* load affected only glucose content. These findings demonstrate that both diet composition and the gut microbiome independently and interactively shape key aspects of host metabolism, although their effects varied depending on the specific nutritional parameter.

**Fig 4:**
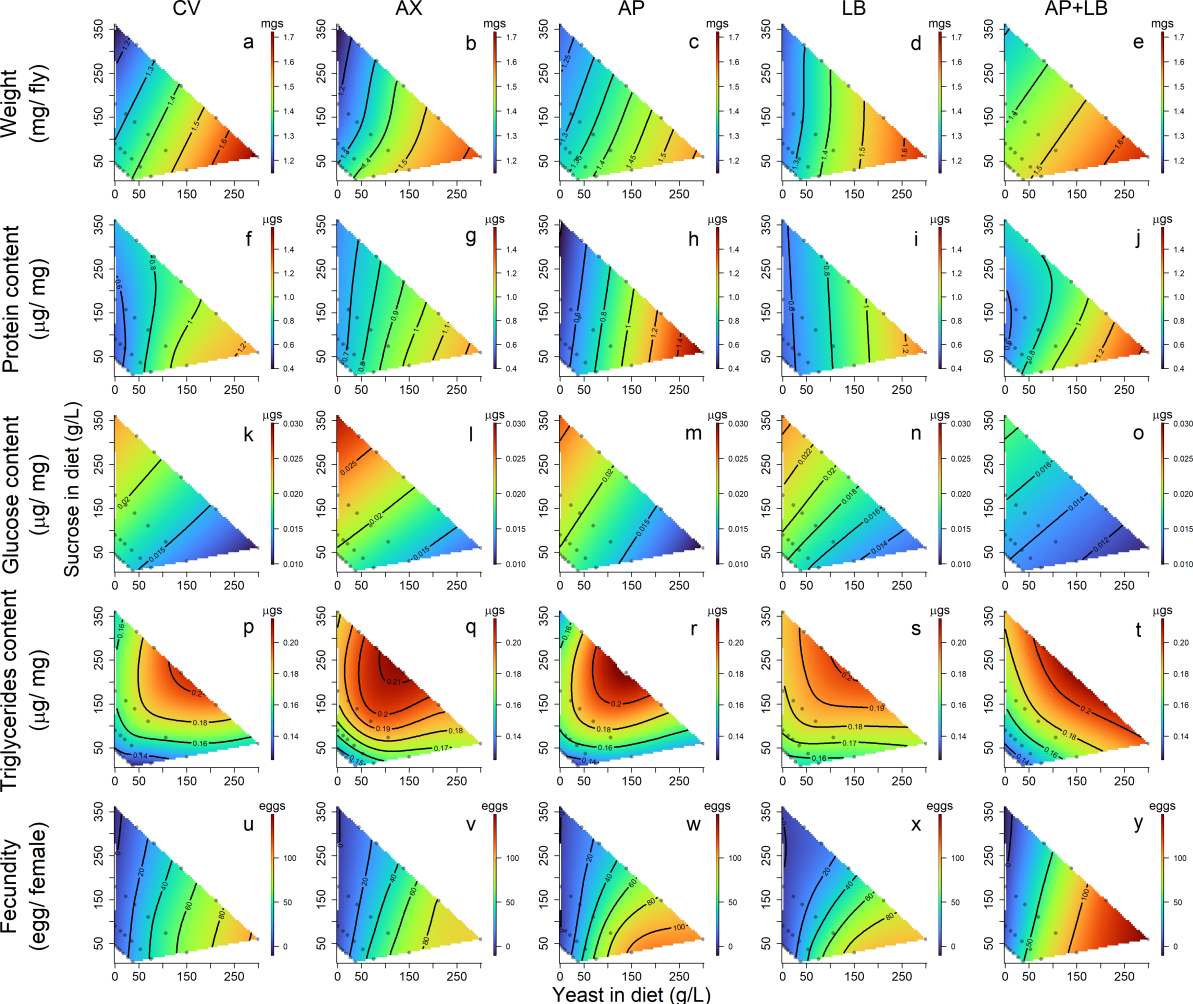
Effects of dietary yeast and sucrose concentrations on body weight (**a–e**), nutritional indices: protein (**f–j**), glucose (**k–o**), triglyceride (**p–t**), and fecundity (**u–y**) of flies with different microbiome compositions. CV: conventional flies; AX: axenic flies; AP: gnotobiotic flies with *Acetobacter pasteurianus*; LB: gnotobiotic flies with *Levilactobacillus brevis*; AP + LB: gnotobiotic flies with both *A. pasteurianus* and *L. brevis*.

To further explore how variations in dietary yeast, sucrose content, and microbiome composition drive the observed patterns in host nutritional status, we utilized a thin-plate spline model to visualize the response surfaces of various nutritional indices. Across all microbiome treatment groups, fly body weight exhibited a positive correlation with dietary yeast levels and a negative correlation with dietary sucrose levels (Fig. 4a through e, and first column in Fig. 6a through e). The highest and lowest weights were observed in CV and AX flies, respectively (Fig. 4a and b). Among the microbiome treatment groups, the LB group showed the strongest positive correlation between dietary yeast and body weight (Pearson’s correlation coefficient, r = 0.76, Fig. 6d), suggesting that *L. brevis* promotes the host’s weight gain in response to protein nutrition.

Fly protein content increased with the dietary yeast (Fig. 4f through j and the second column in Fig. 6a through e). This positive relationship was most pronounced in the AP group (Fig. 4h), with a correlation coefficient (r) of 0.70, followed by the CV group (r = 0.62), AP + LB groups (r = 0.61), the LB group (r = 0.49), and was weakest in the AX group (r = 0.43) (Fig. 6a through e). These findings suggest that *A. pasteurianus* is particularly effective at promoting host protein assimilation.

Glucose levels in the flies showed a significant positive correlation with dietary sucrose and a negative correlation with dietary yeast (Fig. 4k through o and the third column in Fig. 6a through e). Notably, flies in the AX (axenic) group exhibited significantly elevated glucose levels on yeast-deficient, sucrose-rich diets compared with the other groups (Fig. 4l). This suggests that the presence of bacteria in the flies facilitates sugar utilization, leading to reduced glucose accumulation, particularly in the AP + LB group, where the impact was most pronounced (Fig. 4o). Additionally, the flies’ glucose content showed a stronger negative correlation with *A. pasteurianus* load than with *L. brevis* load in the mono-associated groups (Fig. 6c and d). However, the opposite trends were observed in the CV and AP + LB groups (Fig. 6a and e), indicating that microbial interactions modulate sugar metabolism differently compared with single-species associations.

Fly TAG levels showed a general positive relationship with dietary sucrose level (the fourth column in Fig. 6a through e), with the strongest correlation observed in the CV group (r = 0.42) and the weakest in the LB group (r = 0.26). The Y:S ratio of the diet emerged as a key determinant of fly TAG levels, with the highest TAG concentrations occurring at a Y:S ratio of 1:1.6 and increasing further with higher overall diet concentrations (Fig. 4q through t). Among the microbiome treatment groups, the highest TAG levels were observed in the AX group (Fig. 4q).

### Microbiome × diet interactions on fecundity

We assessed fecundity by measuring the number of eggs laid per fly to determine how varying dietary yeast and sucrose content and microbiome compositions influenced reproductive output. Bayesian hierarchical models revealed that diet accounted for 16.3 and 11.2 times more variance in fecundity compared to the microbiome and microbiome × diet interaction, respectively, suggesting a more dominant effect of dietary composition on reproductive performance (Fig. 3a). Within the dietary influence, yeast exhibited the highest relative importance (43%), substantially exceeding that of sucrose (3%), as shown in the fifth column of Fig. 3b. This was followed by the Y:S ratio and its interaction with total dietary concentration, with relative importance values of 24% and 25% of the model’s explained variance, respectively.

A strong and consistent positive correlation was observed between dietary yeast content and fecundity across all microbiome groups (Fig. 4u through y), with Pearson’s coefficients ranging from 0.77 (LB group) to 0.85 (CV group) (the fifth column in Fig. 6a through e). In contrast, sucrose content did not show a significant relationship with fecundity. The gut microbiome also exerted a stimulatory effect on fecundity, although its influence was weaker compared with that of dietary yeast. Among the microbiome groups, *A. pasteurianus* had a more pronounced positive effect on fecundity than *L. brevis*. Interestingly, flies in the AP + LB group exhibited the highest fecundity on high-yeast diets (Fig. S3), suggesting that the presence of both AP and LB synergizes with high-yeast diets to maximize reproductive output. These results suggest that dietary yeast, the source of protein, and other micronutrients play a dominant role in driving fecundity, whereas the microbiome exerts a smaller yet measurable influence, potentially through synergistic interactions among microbial species.

### Microbiome × diet interactions on locomotion and sleep

We measured two behavioral indices: average activity count (locomotion) and sleep duration during both daytime and nighttime. Our Bayesian hierarchical modeling analyses demonstrated that diet and microbiome × diet interaction exerted more pronounced influence on both locomotion and sleep compared to the microbiome alone (Fig. 3a). Nonetheless, a substantial proportion of the residual variability observed in the activity counts and sleep duration metrics, ranging from 39.2% to 62.8% during both daytime and nighttime conditions, was attributed to individual differences among the flies, highlighting the considerable degree of inter-individual variability present in the data.

Relative importance analysis revealed that dietary yeast was the most influential predictor overall, especially for nighttime activity (53%) and sleep (48%) (Fig. 3b). For daytime activity and sleep, yeast remained important (27% and 38%, respectively), but the Y:S ratio also contributed substantially (36% and 32%). The interaction term (Conc. × Y:S) accounted for 17%–26% across the behavioral traits. By contrast, dietary sucrose had minimal influence in all models (≤15% for daytime activity and ≤3% for the rest). These patterns suggest that yeast concentration is the dominant factor of locomotor activity and sleep, particularly at night, whereas daytime behaviors are more sensitive to the relative proportion of yeast and sucrose.

**Fig 5:**
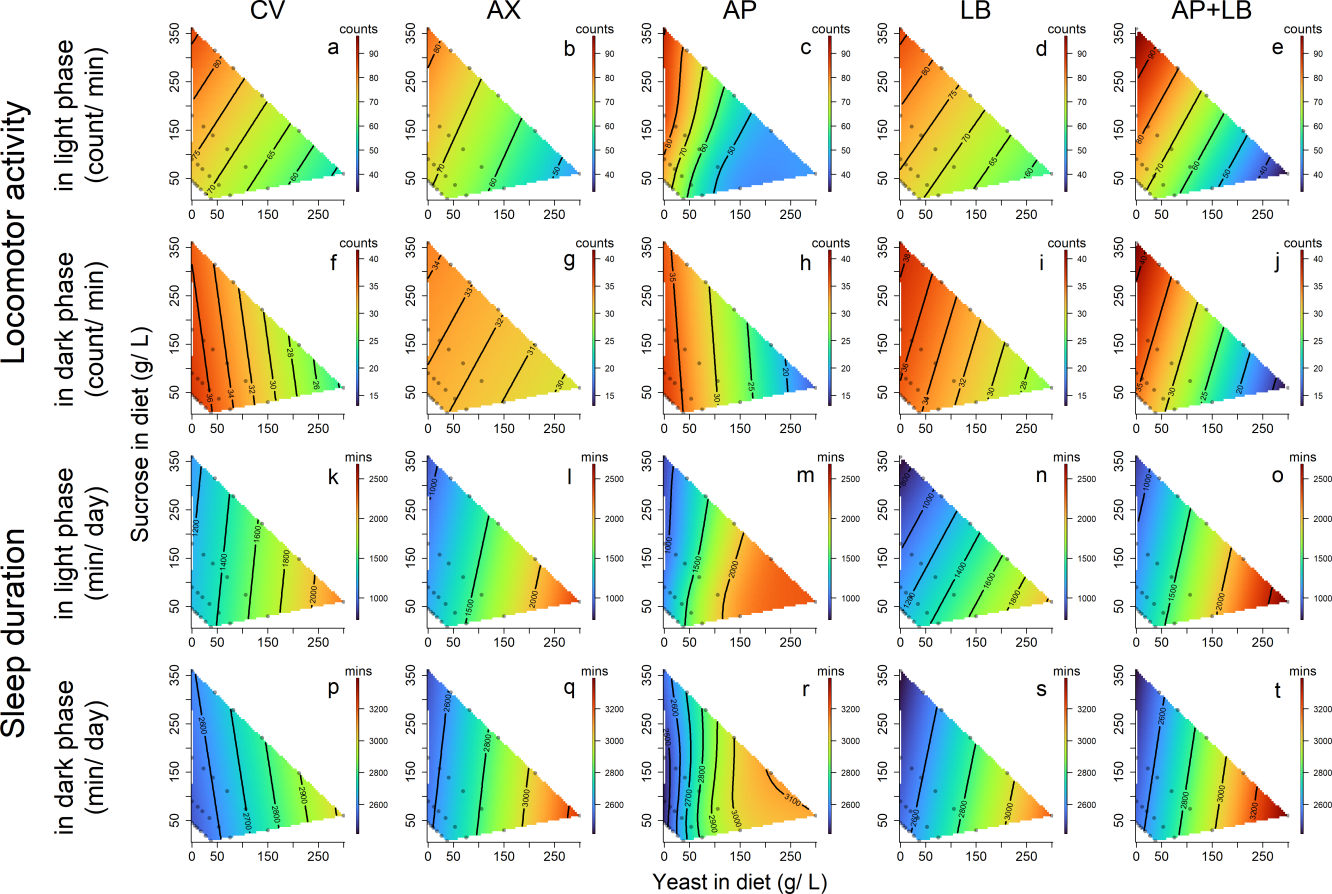
Effects of dietary yeast and sucrose concentrations on locomotor activity in light phase (**a–e**), locomotor activity in dark phase (**f–j**), sleep duration in light phase (**k–o**), and sleep duration in dark phase (**p–t**) of flies with different microbiome compositions. CV: conventional flies; AX: axenic flies; AP: gnotobiotic flies with *A. pasteurianus*; LB: gnotobiotic flies with *L. brevis*; AP + LB: gnotobiotic flies with both *A. pasteurianus* and *L. brevis*. Activity: counts a fly passing through the red beams; Sleep duration: minutes a fly sleeps per day.

Response surface heatmaps indicated that flies fed on high-sucrose, low-yeast diets exhibited the highest levels of locomotor activity during both day and night. This disparity in locomotion was most pronounced in the AP and AP + LB groups (Fig. 5a through e). Sleep duration showed some inverse trends compared with locomotor activity, with shorter durations observed in flies fed on high-sucrose diets and longer durations in those on high-yeast diets (Fig. 5k through t).

**Fig 6:**
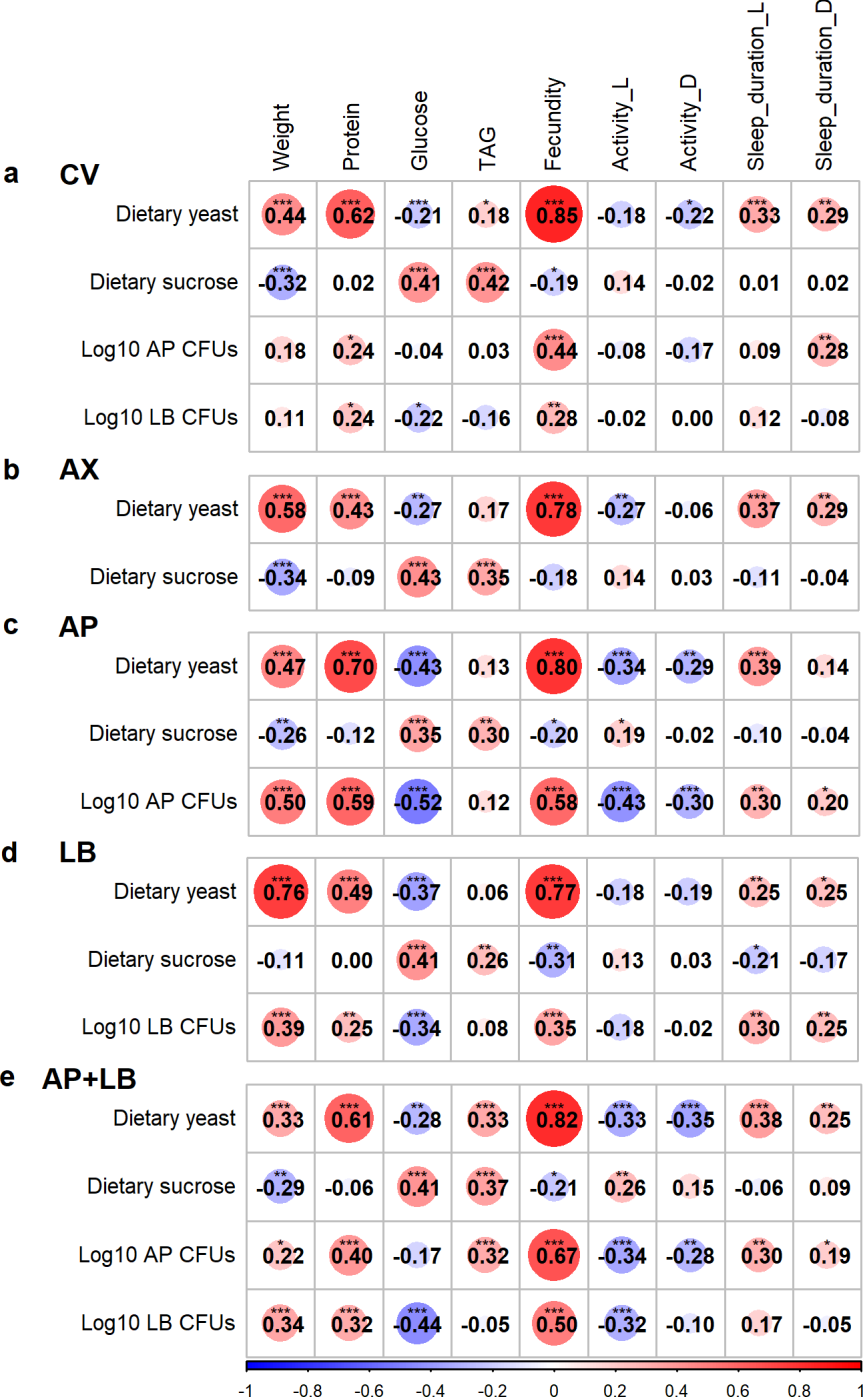
Pearson’s correlation coefficients between dietary nutrients, bacterial loads, and fly traits (body weight, nutritional indices, fecundity, and behavioral parameters). (**a**) CV: conventional flies; (**b**) AX: axenic flies; (**c**) AP: gnotobiotic flies with *Acetobacter pasteurianus*; (**d**) LB: gnotobiotic flies with *Levilactobacillus brevis*; (**e**) AP + LB: gnotobiotic flies with both *A. pasteurianus* and *L. brevis*. Activity: counts a fly passing through the red beams; sleep duration: minutes a fly sleeps per day; L: light phase (daytime); D: dark phase (nighttime). Asterisks indicate statistically significant differences (*** *P* < 0.001, ** *P* < 0.01, *P < 0.05).

Pearson’s coefficients revealed a mild-to-moderate negative relationship between dietary yeast and daytime/nighttime locomotor activity, ranging from −0.34 to −0.18 (the sixth column in Fig. 6), whereas a positive relationship was observed with sleep duration (0.25–0.39; eighth column in Fig. 6). However, for dietary sucrose, the Pearson’s correlation coefficients for these behaviors across microbiome groups were small (r = 0.13–0.26 for daytime locomotor activity and −0.21 to 0.01 for daytime sleep; sixth and eighth columns in Fig. 6), indicating weak linear relationships.

In terms of microbiome effects, the MLR analysis suggested that *A. pasteurianus* load had a greater impact on locomotor activity (sixth and seventh columns in Fig. 3b), with mild-to-moderate negative correlations as indicated by Pearson’s coefficients (ranging from −0.43 in the AP group to −0.08 in the CV group, and from −0.30 in the AP group to −0.17 in the CV group; Fig. 6). Conversely, *L. brevis* load and its interaction with *A. pasteurianus* showed relatively high importance for nighttime sleep duration, contributing 4% and 7% to the explained variance, respectively (the last column in Fig. 3b). These findings indicate that microbial load, particularly that of *A. pasteurianus*, influences locomotor activity, whereas microbial interactions may contribute to the regulation of sleep patterns under specific microbiome conditions.

### Correlation between behavioral and nutritional parameters

**Fig 7:**
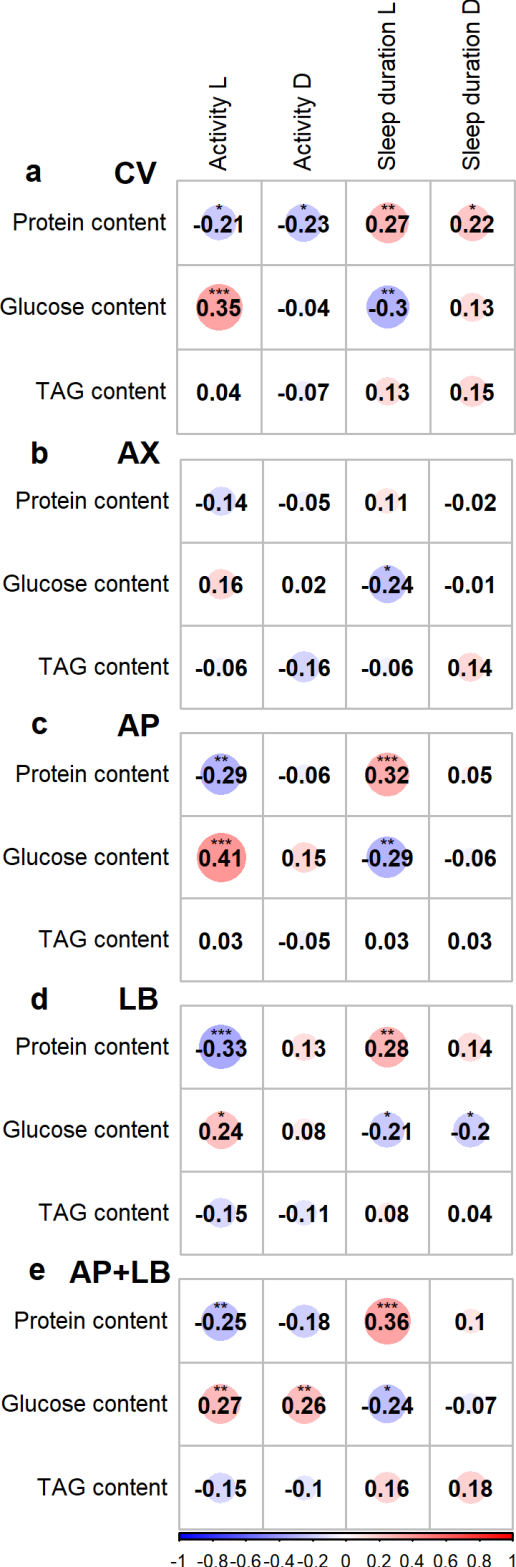
Pearson’s correlation coefficients between fly nutritional indices and behavioral parameters (**a**) CV: conventional flies; (**b**) AX: axenic flies; (**c**) AP: gnotobiotic flies with *A. pasteurianus*; (**d**) LB: gnotobiotic flies with *L. brevis*; (**e**) AP + LB: gnotobiotic flies with both *A. pasteurianus* and *L. brevis*. Metrics: Activity: counts a fly passing through the red beams; sleep duration: minutes a fly sleeps per day; L: light phase (daytime); D: dark phase (nighttime). Asterisks indicate statistically significant differences (*** *P* < 0.001, ** *P* < 0.01, * *P* < 0.05).

We then investigated whether diet-induced changes in nutritional phenotypes were associated with behavioral traits across microbiome treatments, using Pearson’s correlation analysis. Figure 7 illustrates the correlation between locomotor activity, sleep duration, and the three nutritional indices (fly protein, glucose, and TAG contents). A notable observation was that the AX group displayed markedly weaker associations between behavior and nutritional indices compared with all other groups. Among the bacteria-associated groups, we found consistent relationships between protein and glucose levels and daytime behavior. Specifically, fly protein content was negatively correlated with daytime locomotor activity (r ranging from −0.33 in the LB group to −0.21 in the CV group) and positively correlated with daytime sleep duration (r ranging from 0.27 in the CV group to 0.36 in the AP + LB group). In contrast, fly glucose content exhibited the opposite trend, with positive correlations with daytime locomotor activity (r ranging from 0.24 in the LB group to 0.41 in the AP group) and negative correlations with sleep duration (r from −0.21 in the LB group to −0.30 in the CV group). Interestingly, TAG levels generally had much weaker relationships with both locomotor activity and sleep, suggesting a less prominent role in influencing these behavioral patterns. These findings suggest that fly protein and glucose levels may serve as reliable indicators of behavioral tendencies.

## DISCUSSION

By systematically analyzing 120 unique diet-microbiome combinations in *Drosophila*, our results revealed that diet and microbiome play distinct and context-dependent roles in shaping host nutritional profiles. Diet had a stronger influence on fly protein and TAG levels, explaining 4.6 and 1.7 times more variation than the microbiome, respectively, whereas the microbiome effect on glucose levels was comparable with that of diet, each accounting for about one-third of the observed variation. This finding aligns with prior studies that show flies with gut bacteria maintain lower glucose levels than axenic flies, likely due to microbial fermentation of sugars and modulation of host metabolic signaling (3, 8, 34). Diet × microbiome effects were relatively low for glucose and TAG but more pronounced for proteins and weight.

Macronutrient balance has long been recognized as a key determinant of insect nutritional ecology, with numerous studies showing that insects actively regulate their intake of protein and carbohydrates to optimize life-history traits (16, 35–37). In *Drosophila*, Lee demonstrated that lipid reserves vary with dietary protein-to-carbohydrate (P:C) ratio, with higher TAG levels generally occurring on intermediate P:C diets (1:2 and 1:4 P:C), rather than at extremes of high sugar or protein content (38). Our results confirm and extend these observations by showing that fly TAG levels are strongly influenced by the Y:S ratio, with the highest TAG accumulation observed at 1:1.6 (corresponding to a 1:4 P:C). Moreover, the effects of macronutrient balance on TAG levels became more pronounced at higher overall nutrient concentrations, suggesting that nutrient density amplifies the influence of Y:S balance on lipid storage. Importantly, we observed that microbiome composition influences TAG metabolism in a nutrient-dependent manner. Axenic flies exhibited the highest TAG levels at the peak Y:S (1:1.16), whereas bacteria-associated flies showed attenuated TAG accumulation, suggesting that the gut bacteria buffer against excessive fat accumulation. This buffering effect is consistent with previous findings that showed *A. pomorum* mitigates preservative-induced TAG increases (39) and that the presence of gut microbiome reduces lipid accumulation in flies (3, 8). Collectively, these findings highlight that macronutrient balance and gut bacteria coordinately regulate lipid storage.

Dietary yeast also had a superior impact on multiple phenotypic traits, including body weight and fecundity. As yeast is the only source of protein in the fly diet, this observation affirms the fundamental role of protein as a building block for tissue growth and egg production (2, 38, 40). Our results demonstrate that interactions with the gut microbiome added an important layer of complexity to these outcomes. In the study by Yamada et al. (41), which compared axenic flies with gnotobiotic flies associated with the live yeast *Issatchenkia orientalis,* the microbe was found to enhance host nutrition by directly extracting amino acids from the diet for the fly. In our study, using a yeast-sugar diet containing inactive yeast, we demonstrate that gut bacteria not only facilitate protein assimilation, leading to weight gain and increased protein levels in flies, but also fine-tune the host’s response to dietary shifts. *A. pasteurianus* was particularly effective in amplifying the positive effects of dietary yeast on host weight and protein levels compared with axenic flies. Furthermore, *A. pasteurianus* exhibited a clear affinity for yeast-rich diets, with increased abundance corresponding to higher dietary yeast levels in both mono-associated (AP group) and dual-associated (AP + LB group) conditions. This supports and extends the findings by Henry et al. (2), who observed a positive correlation between dietary yeast concentration and fly bacterial load, suggesting that *A. pasteurianus* thrives in protein-rich environments and may play a key role in protein breakdown or absorption, thereby enhancing host protein assimilation.

An intriguing and novel finding is the distinct effects of microbial interactions on protein assimilation and reproductive outcomes. Microbe-driven enhancement of host reproduction has been reported by Suyama et al. (42), where microbes promoted germline stem cell (GSC) proliferation and ovarian cell division. Additionally, metabolic cooperation among commensal bacteria has been shown to support *Drosophila* juvenile growth under nutritional stress (43). Although we did not observe any additive or synergistic effects of *A. pasteurianus* and *L. brevis* on protein assimilation, their combined presence achieved the greatest reproductive output on yeast-rich diets. This indicates that although *A. pasteurianus* may primarily support fecundity through enhanced protein nutrition, *L. brevis* could provide additional benefits that further boost reproductive success, such as the production of lactate (43), other micronutrients (44), or mitochondrial coenzymes (45), warranting further investigation.

Our behavioral data indicate that diet exerts a dominant influence on both locomotion and sleep. High-yeast diets consistently lead to increased sleep duration, in line with the notion that high-protein dietary interventions, or specific amino acids, may promote sleep homeostasis and improve sleep quality (46–48). Conversely, flies on high-sucrose, low-yeast diets exhibited hyperactivity and reduced sleep duration during both daytime and nighttime. These findings are consistent with the ecological principles in which limited protein availability prompts increased foraging activities and behavioral adjustments to meet nutritional needs (49–52). In *D. melanogaster*, protein deprivation has been shown to activate specific neural circuits that enhance protein-seeking behavior. In mated female flies, protein scarcity was found to activate a specific dopamine circuit that promotes protein-seeking behavior and reduces sugar intake. This neural adaptation ensures that protein-deprived animals prioritize seeking and consuming protein over other nutrients (50). Interestingly, our finding contrasts with the study of Catterson et al. (53), which showed that a high-sucrose diet reduces total sleep only in male but not in female flies.

The role of the microbiome in regulating sleep and locomotion has been widely debated, with conflicting results across studies. Selkrig et al. (54) observed negligible microbiome effects on sleep and locomotion. Silva et al. (55), in contrast, reported that axenic flies exhibited hyperactivity and extended sleep durations, with the microbiome modulating sleep rebound following deprivation. Similarly, Schretter et al. (21) observed hyperactivity in axenic flies and demonstrated that normal locomotor activity could be restored by mono-association with *L. brevis*. These discrepancies may be explained by the differences in dietary compositions, as highlighted by our findings. Notably, diet × microbiome interactions explained more variance in locomotor activity and sleep than microbiome alone, and, in some cases, even exceeded the effect of diet. This contrasts with nutritional traits, where interaction effects were relatively small. For instance, the presence of *Acetobacter* significantly amplified the contrast in locomotor activity between high-sugar, low-yeast and low-sugar, high-yeast diets. Additionally, the combined presence of *Acetobacter* and *Levilactobacillus* further enhanced sleep duration in flies on high-yeast diets. Together, these results underscore the complex and interactive effects of diet and microbiome on behavior, suggesting that the microbiome’s influence on locomotion and sleep is variable and contingent on the dietary conditions.

Despite the new insights provided by our study, it is important to acknowledge several limitations. Although the geometric framework was rigorously applied by systematically varying dietary yeast and sucrose, the impact of dietary fat and micronutrients was not explicitly tested. Additionally, our study focused primarily on female flies, and potential sex differences in the diet-microbiome interaction remain to be explored. Furthermore, although the use of the lab-adapted Canton-S strain is advantageous for its genetic homogeneity and widespread use in previous fly microbiome, nutrition, and physiology studies (1, 16, 56), lab strains may not fully recapitulate the genetic diversity and environmental adaptations of wild *Drosophila* populations. This controlled approach was practical to manage the complexity of testing 120 nutrient-microbiome combinations, but highlights the need for future studies on multiple genotypes and wild flies. Nonetheless, this study advances the fundamental understanding of how specific diet-microbiome interactions shape host metabolism, reproduction, and behavior. Further research will focus on the molecular pathways through which diet-microbiome interactions drive specific phenotypic outcomes and translate these insights into other animal models to gain broader contexts.

## Data Availability

The datasets and R scripts used and/or analyzed in this study have been deposited in the GitHub repositories “Nutrient-microbiome-interactions-on-Drosophila” and “Trikinetic_DAM5H”. The 16S rRNA gene sequences of the bacterial isolates used in this study have been deposited in NCBI GenBank under accession numbers PQ451486 and PQ451487.

## SUPPLEMENTAL MATERIAL

Supplemental figure legends mbio.02480-25-s0001.docx Legends for Figures S1-S3.

Figure S1 mbio.02480-25-s0002.tif Experimental scheme of the data analysis process.

Figure S2 mbio.02480-25-s0003.tif Fit plot for CFUs of *A. pasteurianus* and *L. brevis* in gnotobiotic flies.

Figure S3 mbio.02480-25-s0004.tif Fecundity of flies on 24 diets across five microbiota groups.

Supplemental tables mbio.02480-25-s0005.docx Tables S1-S4.

ASM does not own the copyrights to Supplemental Material that may be linked to, or accessed through, an article. The authors have granted ASM a non-exclusive, world-wide license to publish the Supplemental Material files. Please contact the corresponding author directly for reuse.
