# Supplementary material for: Systematic analysis of nutrient-microbiome interactions and their effects on host phenotypes in Drosophila
Source: mBio. 2025 Nov 5;16(12):e02480-25. doi: 10.1128/mbio.02480-25 (PMC12691620; doi:10.1128/mbio.02480-25)
Supplement: Supplemental figure legends — Legends for Figures S1-S3. [file mbio.02480-25-s0001.docx]

**Supplemental figure legends**

**Figure S1.** Experimental scheme of the data analysis process. (a) To identify whether the microbiome, diet, or their interaction (diet × microbiome) was the dominant factor influencing nutritional, reproductive, and behavioral traits, we first employed Bayesian hierarchical generalized linear mixed models (GLMMs) to estimate the proportion of variance attributable to broad microbial and dietary categories. (b) To evaluate the individual contributions of specific sub-level factors—dietary yeast, sucrose, yeast-to-sucrose ratio, total diet concentration, and colony-forming units (CFUs) of *A. pasteurianus* (AP) and *L. brevis* (LB)—in explaining variation of outcome variables, we decomposed the total model R^2^ from multiple linear regression models to quantify their influence on nutritional, fecundity and behavioral traits. (c) To further investigate trait patterns, Pearson’s correlation analysis was conducted to assess linear relationships among traits within each microbiome group. (d) Given the potential impact of nutritional profiles on behavioral outcomes, we also analyzed correlations between nutritional and behavioral traits under each microbiome condition.

**Figure S2.** Fit plot for Colony-forming units (CFUs) of *Acetobacter pasteurianus* and *Levilactobacillus brevis* in (a) the gnotobiotic fly with *A. pasteurianus* (AP)*,* (b) gnotobiotic fly with *L. brevis* (LB), (c) gnotobiotic fly with two species with both *A. pasteurianus* and *L. brevis* (AP+LB), and (d) the conventional fly (CV) fed on varying sucrose diets. The red lines indicate CFUs of *A. pasteurianus.* The blue lines indicate the CFUs of *L. brevis.* Lines are fitted using locally estimated scatterplot smoothing. The gray shade area represents the 95% confidence interval. *R*: the correlation coefficient; *R*^2^: The coefficient of determination, *p*: P-value of the correlation coefficient.

**Figure S3.** Fecundity of flies on 24 diets cross five microbiota groups. Statistically significant differences are indicated by different colors and letters (Kruskal–Wallis test followed by Dunn’s post hoc test, *p* < 0.05).
