# Supplementary figures and images for: Systematic analysis of nutrient-microbiome interactions and their effects on host phenotypes in Drosophila
Source: mBio. 2025 Nov 5;16(12):e02480-25. doi: 10.1128/mbio.02480-25 (PMC12691620; doi:10.1128/mbio.02480-25)

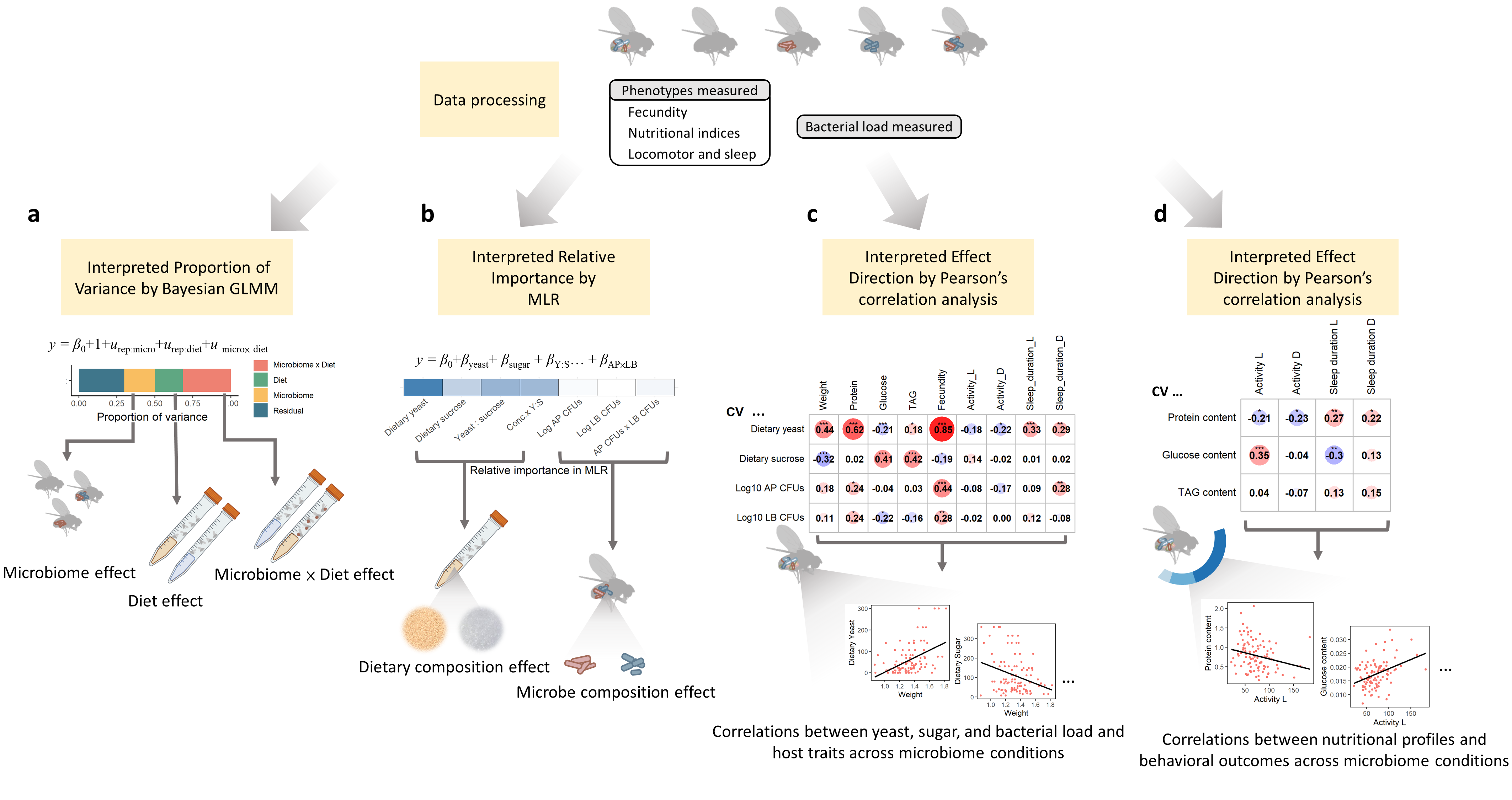

Supplement: Figure S1 — Experimental scheme of the data analysis process. [file mbio.02480-25-s0002.tif]

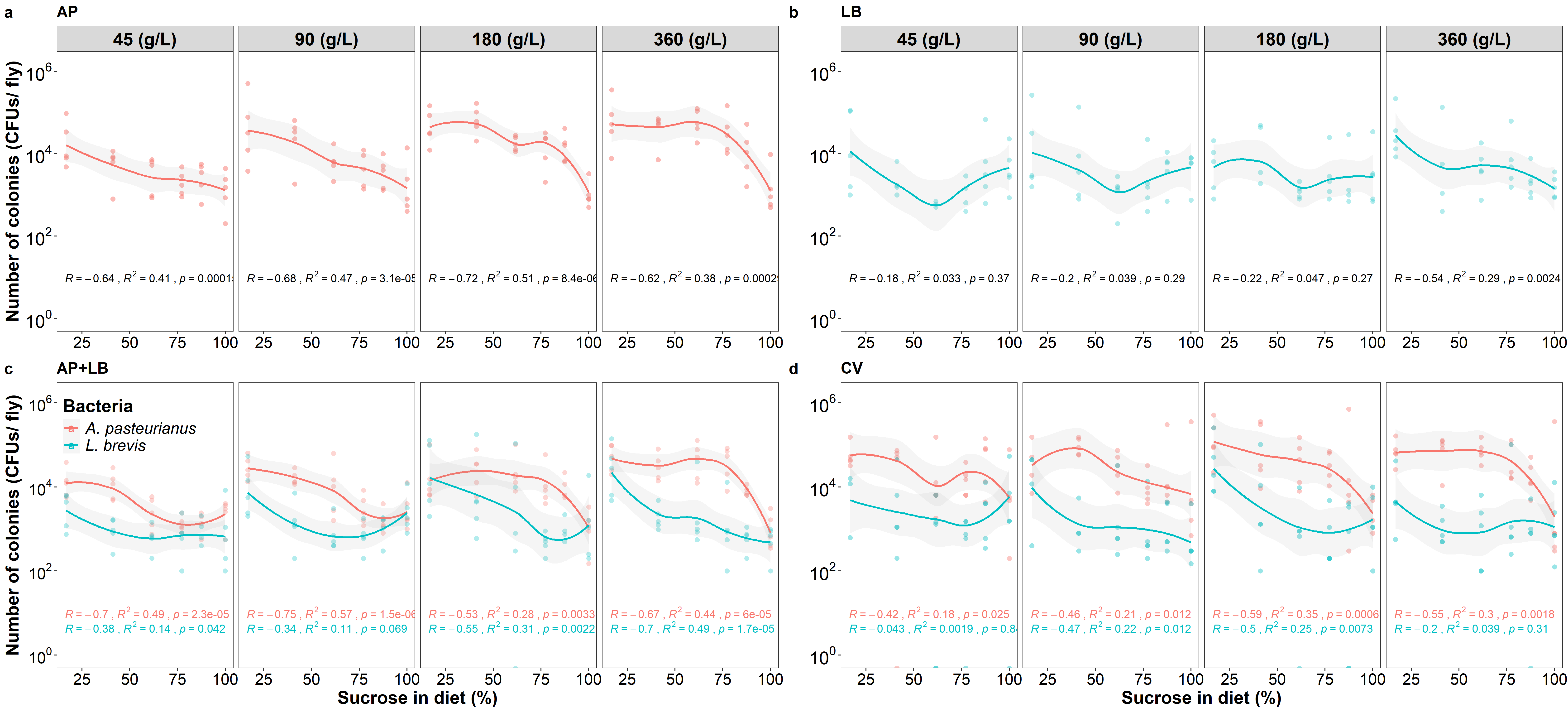

Supplement: Figure S2 — Fit plot for CFUs of A. pasteurianus and L. brevis in gnotobiotic flies. [file mbio.02480-25-s0003.tif]

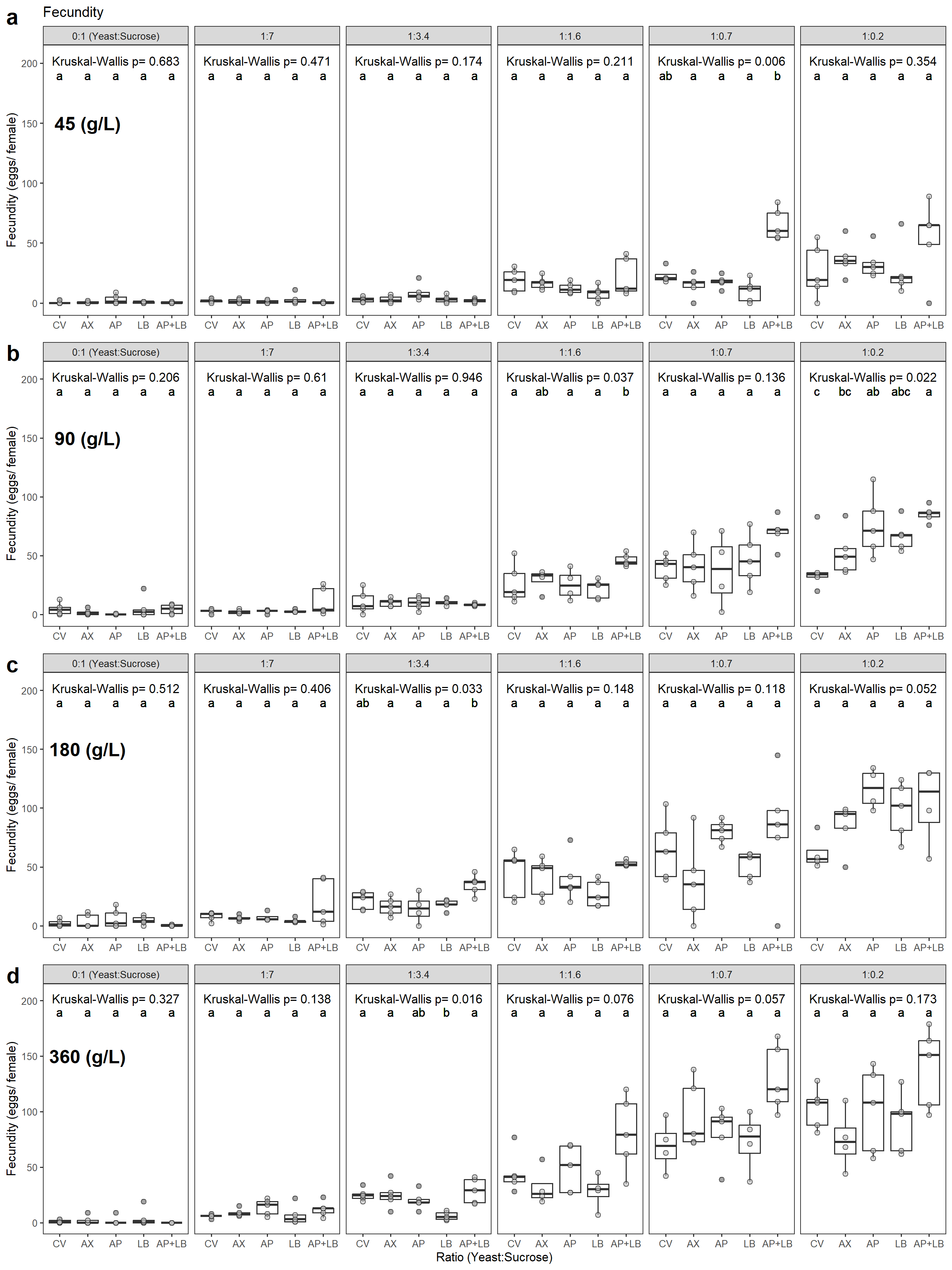

Supplement: Figure S3 — Fecundity of flies on 24 diets across five microbiota groups. [file mbio.02480-25-s0004.tif]
