# Supplementary material for: Systematic analysis of nutrient-microbiome interactions and their effects on host phenotypes in Drosophila
Source: mBio. 2025 Nov 5;16(12):e02480-25. doi: 10.1128/mbio.02480-25 (PMC12691620; doi:10.1128/mbio.02480-25)
Supplement: Supplemental tables — Tables S1-S4. [file mbio.02480-25-s0005.docx]

**Supplementary Table 1.** Coefficients (± SE) and p-values for the effects of dietary yeast, sucrose, and their interaction on bacterial CFUs in flies, based on linear models

|  | **Dietary yeast** | | **Dietary sucrose** | | **Dietary yeast: dietary sucrose** | | **Concentration level ×Dietary yeast: dietary sucrose** | |
| --- | --- | --- | --- | --- | --- | --- | --- | --- |
|  | Estimate | *p*-value | Estimate | *p*-value | Estimate | *p*-value | Estimate | *p*-value |
| AP CFUs^a^ | 0.0081  (0.0030) | 0.0068  (**)^d^ | -0.0005  (0.0011) | 0.6096 | 0.0806  (0.0878) | 0.3591 | -0.0013  (0.0007) | 0.0522 |
| LB CFUs^b^ | -0.0009  (0.0025) | 0.7139 | 0.0003  (0.0009) | 0.7315 | 0.1015  (0.0742) | 0.1718 | 0.0002  (0.0006) | 0.6924 |
| Total CFUs^c^ | 0.0072  (0.0041) | 0.0804 | -0.0002  (0.0014) | 0.8726 | 0.1821  (0.1208) | 0.1321 | -0.0011  (0.0009) | 0.2420 |

^a^AP CFUs: bacterial load of Acetobacter pasteurianus in flies.
^b^LB CFUs: bacterial load of Levilactobacillus brevis in flies.
^c^Total CFUs: combined bacterial load of A. pasteurianus and L. brevis in flies.

^d^Asterisks indicate statistically significant differences -*** *p* < 0.001, ** *p* <0.01, * *p* <0.05.
CFU data were log-transformed prior to model fitting.

**Supplementary Table 2.** Relative contributions (with confidence intervals) from linear models estimating the effects of dietary yeast, sucrose, and their interaction on bacterial CFUs in flies, based on *R*^2^ values

|  | **Dietary yeast** | **Dietary sucrose** | **Dietary yeast: dietary sucrose** | **Concentration level ×Dietary yeast: dietary sucrose** |
| --- | --- | --- | --- | --- |
| AP CFUs^a^ | 0.6152  (0.1801-0.6831) | 0.0207  (0.0093-0.3844) | 0.1114  (0.0323-0.4853) | 0.2526  (0.1551-0.3936) |
| LB CFUs^b^ | 0.1316  (0.0491-0.4473) | 0.0463  (0.0202-0.4526) | 0.5563  (0.1041-0.7472) | 0.2658  (0.0979-0.5472) |
| Total CFUs^c^ | 0.4012  (0.0922-0.6368) | 0.0288  (0.0119-0.4117) | 0.3412  (0.056-0.6264) | 0.2288  (0.1335-0.3681) |

^a^AP CFUs: bacterial load of Acetobacter pasteurianus in flies.
^b^LB CFUs: bacterial load of Levilactobacillus brevis in flies.
^c^Total CFUs: combined bacterial load of A. pasteurianus and L. brevis in flies.
CFU values were log-transformed prior to model fitting.
Relative contributions were normalized to sum to 1.
Confidence intervals were estimated via bootstrap resampling (400 replicates).
No significant differences were detected within each row, based on non-overlapping confidence intervals at p < 0.05.

**Supplementary Table 3.** Proportion of variance (with confidence intervals) explained by diet and microbiome factors for the different nutritional indices and behavioral parameters studied on Bayesian hierarchical models

| **Variables** |  | **Proportion** | **Mean** | **mcse**^a^ | **SD** | **2.50%** | **97.50%** | **n_eff**^b^ | **Rhat**^c^ |
| --- | --- | --- | --- | --- | --- | --- | --- | --- | --- |
| Log CFU AP^d^ | Microbiome x Diet | 0.0083 | 0.0343 | 0.0054 | 0.0389 | 0.0001 | 0.1267 | 52 | 1.0606 |
|  | Diet | 0.0178 | 0.0737 | 0.0004 | 0.0170 | 0.0441 | 0.1100 | 1680 | 1.0018 |
|  | Microbiome | 0.9299 | 3.8476 | 0.0447 | 1.0382 | 2.2390 | 6.1725 | 538 | 1.0043 |
|  | Residual | 0.0440 | 0.1819 | - | - | - | - | - | - |
| Log CFU LB^e^ | Microbiome x Diet | 0.0233 | 0.0703 | 0.0083 | 0.0596 | < 0.0001 | 0.2083 | 52 | 1.0700 |
|  | Diet | 0.0142 | 0.0430 | 0.0005 | 0.0165 | 0.0129 | 0.0781 | 953 | 1.0029 |
|  | Microbiome | 0.8772 | 2.6503 | 0.0269 | 0.7496 | 1.5996 | 4.4792 | 777 | 1.0022 |
|  | Residual | 0.0853 | 0.2578 | - | - | - | - | - | - |
| Fecundity | Microbiome x Diet | 0.0664 | 83.7466 | 16.3959 | 68.8360 | 0.1032 | 227.9178 | 18 | 1.3112 |
|  | Diet | 0.7415 | 934.6206 | 4.6385 | 134.6395 | 711.7748 | 1249.0904 | 843 | 1.0025 |
|  | Microbiome | 0.0454 | 57.2538 | 0.7843 | 23.1056 | 24.9239 | 110.9391 | 868 | 1.0088 |
|  | Residual | 0.1466 | 184.8418 | - | - | - | - | - | - |
| Weight | Microbiome x Diet | 0.2025 | 0.0057 | 0.0009 | 0.0039 | < 0.0001 | 0.0126 | 18 | 1.2929 |
|  | Diet | 0.3156 | 0.0089 | < 0.0001 | 0.0015 | 0.0061 | 0.0124 | 1330 | 1.0072 |
|  | Microbiome | 0.1828 | 0.0051 | 0.0003 | 0.0020 | 0.0026 | 0.0097 | 39 | 1.1078 |
|  | Residual | 0.2990 | 0.0084 | - | - | - | - | - | - |
| Protein | Microbiome x Diet | 0.1280 | 0.0167 | 0.0025 | 0.0155 | < 0.0001 | 0.0507 | 38 | 1.2239 |
|  | Diet | 0.4045 | 0.0528 | 0.0002 | 0.0088 | 0.0374 | 0.0716 | 1422 | 1.0008 |
|  | Microbiome | 0.0884 | 0.0115 | 0.0001 | 0.0047 | 0.0047 | 0.0231 | 1269 | 1.0021 |
|  | Residual | 0.3791 | 0.0495 | - | - | - | - | - | - |
| Glucose | Microbiome x Diet | 0.0748 | < 0.0001 | < 0.0001 | < 0.0001 | < 0.0001 | < 0.0001 | 22 | 1.2504 |
|  | Diet | 0.3368 | < 0.0001 | < 0.0001 | < 0.0001 | < 0.0001 | < 0.0001 | 1432 | 1.0034 |
|  | Microbiome | 0.3307 | < 0.0001 | < 0.0001 | < 0.0001 | < 0.0001 | < 0.0001 | 1107 | 1.0042 |
|  | Residual | 0.2577 | < 0.0001 | - | - | - | - | - | - |
| TAG | Microbiome x Diet | 0.0697 | 0.0002 | < 0.0001 | 0.0002 | < 0.0001 | 0.0006 | 58 | 1.0282 |
|  | Diet | 0.3633 | 0.0008 | < 0.0001 | 0.0001 | 0.0006 | 0.0011 | 1388 | 1.0015 |
|  | Microbiome | 0.2120 | 0.0005 | < 0.0001 | 0.0002 | 0.0002 | 0.0009 | 485 | 1.0093 |
|  | Residual | 0.3550 | 0.0008 | - | - | - | - | - | - |
| Activity L^f^ | Microbiome x Diet | 0.2428 | 185.6287 | 18.4788 | 104.4050 | 0.1859 | 360.6279 | 32 | 1.1638 |
|  | Diet | 0.2105 | 160.9887 | 1.3455 | 41.6763 | 92.1317 | 255.6324 | 959 | 1.0033 |
|  | Microbiome | 0.1474 | 112.7125 | 1.7285 | 52.4726 | 39.1919 | 240.6033 | 922 | 1.0053 |
|  | Residual | 0.3993 | 305.3009 | - | - | - | - | - | - |
| Activity D | Microbiome x Diet | 0.2679 | 64.2858 | 20.9444 | 42.2039 | 0.3233 | 143.8525 | 4 | 1.5062 |
|  | Diet | 0.2412 | 57.8634 | 0.5195 | 15.2222 | 32.3915 | 91.9573 | 859 | 1.0073 |
|  | Microbiome | 0.0992 | 23.8038 | 0.5036 | 13.6910 | 4.2384 | 57.7491 | 739 | 1.0030 |
|  | Residual | 0.3917 | 93.9734 | - | - | - | - | - | - |
| Sleep duration L^g^ | Microbiome x Diet | 0.1877 | 314.1032 | 41.9678 | 229.6285 | 0.3472 | 772.0693 | 30 | 1.1542 |
|  | Diet | 0.3530 | 590.6749 | 3.8547 | 122.0192 | 382.2205 | 851.2743 | 1002 | 1.0055 |
|  | Microbiome | 0.0087 | 14.5861 | 1.0141 | 25.0007 | 0.0087 | 86.5326 | 608 | 1.0076 |
|  | Residual | 0.4505 | 753.7219 | - | - | - | - | - | - |
| Sleep duration D | Microbiome x Diet | 0.1364 | 1211.3953 | 775.5874 | 1868.6418 | 4.0953 | 5824.5328 | 6 | 1.3614 |
|  | Diet | 0.1624 | 1443.0792 | 36.9460 | 460.1186 | 665.5313 | 2439.4109 | 155 | 1.0356 |
|  | Microbiome | 0.0732 | 650.7225 | 11.7442 | 326.2913 | 145.7727 | 1409.6169 | 772 | 1.0035 |
|  | Residual | 0.6280 | 5579.2032 | - | - | - | - | - | - |

**^a^ MCSE** refers to the **Monte Carlo standard error**, which estimates the uncertainty due to finite sampling in Bayesian inference.

**^b^** n_eff denotes the **effective sample size**, reflecting the number of independent draws equivalent to the autocorrelated MCMC samples.

**^c^** Rhat assesses convergence across Markov chains, where values close to 1 indicate good convergence.

^d^CFUs of AP: bacteria load of *Acetobacter pasteurianus* in flies*.*

^e^CFUs of LB: bacteria load of *Lactobacillus brevis* in flies*.*

^f^Activity: counts a fly passing through the red beams.

^g^Sleep duration: minutes a fly sleeps per day.

CFU data were log-transformed prior to model fitting.

Relative contributions were normalized to sum to 1.

Confidence intervals were generated using bootstrap resampling with 400 replicates.

**Supplementary Table 4.** Relative contributions (with confidence intervals) from linear models estimating the effects of dietary yeast, sugar, and bacterial CFUs on nutritional indices, activity, and sleep duration in flies, based on R² values

|  | **Dietary yeast** | | **Dietary sugar** | | **Dietary yeast: dietary sugar** | | **Concentration level ×Dietary yeast: dietary sugar** | |
| --- | --- | --- | --- | --- | --- | --- | --- | --- |
| Fecundity | 0.4258  (0.3707-0.4772) | a | 0.0331  (0.0216-0.0481) | def | 0.2421  (0.1977-0.2919) | bc | 0.2513  (0.2315-0.275) | bc |
| Weight | 0.3691  (0.2722-0.4578) | ab | 0.1368  (0.0766-0.2138) | bcd | 0.2203  (0.1398-0.3122) | abcd | 0.2056  (0.1686-0.2399) | bcd |
| Protein content | 0.3618  (0.2941-0.4177) | ab | 0.0229  (0.0126-0.0583) | def | 0.2723  (0.2071-0.3375) | abc | 0.2896  (0.2481-0.3241) | bc |
| Glucose  content | 0.0842  (0.0489-0.1219) | cdefg | 0.2678  (0.1499-0.3746) | ab | 0.2806  (0.202-0.3609) | ab | 0.1009  (0.0768-0.1282) | cdef |
| TAGs content | 0.3496  (0.2597-0.4409) | abc | 0.2267  (0.1228-0.3382) | abcd | 0.2494  (0.1502-0.3408) | abcd | 0.1439  (0.107-0.1932) | cd |
| Activity (light) | 0.2711  (0.1272-0.3978) | abcd | 0.1458  (0.0348-0.3019) | abcde | 0.3595  (0.207-0.5033) | abc | 0.173  (0.1268-0.2105) | cd |
| Activity (dark) | 0.5272  (0.3159-0.6215) | a | 0.0181  (0.0083-0.1376) | cdefg | 0.1702  (0.0483-0.3127) | bcd | 0.2271  (0.1634-0.25) | bc |
| Sleep (light) | 0.3821  (0.205-0.5205) | abc | 0.0313  (0.01-0.081) | defg | 0.3179  (0.1533-0.4927) | abc | 0.2592  (0.1747-0.3845) | abc |
| Sleep (dark) | 0.479  (0.2299-0.6071) | ab | 0.021  (0.0077-0.1211) | defg | 0.1617  (0.057-0.3132) | abcde | 0.2233  (0.1463-0.339) | bcd |

**Supplementary Table 4.** Relative contributions (with confidence intervals) from linear models estimating the effects of dietary protein, carbohydrate, and bacterial CFUs on nutritional indices, activity, and sleep duration in flies, based on R² values (continued)

|  | **AP CFUs^a^** | | **LB CFUs^b^** | | **AP CFUs × LB CFUs** | | **Proportion of variance explained by model (%)** |
| --- | --- | --- | --- | --- | --- | --- | --- |
| Fecundity | 0.0211  (0.0076-0.0407) | defg | 0.0054  (0.0017-0.016) | fg | 0.0212  (0.0079-0.0443) | def | 71.56 |
| Weight | 0.0222  (0.0038-0.06) | efg | 0.0106  (0.0024-0.0363) | fg | 0.0355  (0.0072-0.0846) | ef | 33.95 |
| Protein content | 0.0113  (0.0037-0.0308) | efg | 0.007  (0.0028-0.0226) | efg | 0.0351  (0.01-0.0782) | def | 38.38 |
| Glucose  content | 0.0705  (0.0287-0.1291) | cdefg | 0.0979  (0.0476-0.1693) | cdefg | 0.0981  (0.0571-0.146) | cdefg | 32.91 |
| TAGs content | 0.0202  (0.003-0.0692) | efg | 0.0034  (0.0014-0.0341) | efg | 0.0068  (0.0021-0.0383) | efg | 27.17 |
| Activity (light)^c^ | 0.0266  (0.0016-0.1063) | defg | 0.0179  (0.0019-0.0909) | efg | 0.0061  (0.0026-0.0445) | efg | 14.30 |
| Activity (dark) | 0.03  (0.0036-0.1596) | cdefg | 0.0197  (0.0019-0.1329) | cdefg | 0.0077  (0.0035-0.0728) | defg | 7.91 |
| Sleep (light)^d^ | 0.0013  (0.0014-0.0622) | defg | 0.0051  (0.0015-0.0949) | defg | 0.0032  (0.0017-0.0492) | defg | 14.87 |
| Sleep (dark) | 0.0121  (0.0031-0.0877) | defg | 0.0377  (0.003-0.2155) | bcdefg | 0.0652  (0.0055-0.178) | cdefg | 7.22 |

^a^CFUs of AP: bacteria load of *Acetobacter pasteurianus* in flies*.*

^b^CFUs of LB: bacteria load of *Lactobacillus brevis* in flies*.*

^c^Activity: counts a fly passing through the red beams.

^d^Sleep duration: minutes a fly sleeps per day.

CFU data were log-transformed prior to model fitting.

Relative contributions were normalized to sum to 1.

Confidence intervals were generated using bootstrap resampling with 400 replicates.

Different letters indicate statistically significant differences within each row, determined by non-overlapping confidence intervals at *p*<0.05.
